# Flow-cytometry-based physiological characterisation and transcriptome analyses reveal a mechanism for reduced cell viability in yeast engineered for increased lipid content

**DOI:** 10.1186/s13068-019-1435-6

**Published:** 2019-04-23

**Authors:** Huadong Peng, Lizhong He, Victoria S. Haritos

**Affiliations:** 1 Department of Chemical Engineering, Monash University, Clayton, VIC 3800 Australia; 2 Present Address: Department of Bioengineering, Imperial College London, London, SW7 2AZ UK

**Keywords:** Metabolic engineering, Triacylglycerol, Reactive oxygen species, Mitochondrial membrane potential, Transcriptome analysis

## Abstract

**Background:**

Yeast has been the focus of development of cell biofactories for the production of lipids and interest in the field has been driven by the need for sustainably sourced lipids for use in a broad range of industrial applications. Previously, we reported a metabolic engineering strategy for enhanced lipid production in yeast which delivered high per-cell lipid but with low cell growth and compromised physiology. To investigate the relationship between lipid engineering and cellular physiological responses and to identify further metabolic engineering targets, we analysed transcriptomes and measured cell physiology parameters in engineered strains.

**Results:**

In the engineering strategy, the central carbon pathway was reprogrammed to provide more precursors for lipid production and lipid accumulation and sequestration steps were enhanced through the expression of heterologous genes. Genes coding for enzymes within the pentose phosphate, beta-oxidation pathways, ATP and NADPH biosynthesis had lower transcript levels in engineered cells. Meanwhile, flow-cytometry analysis of fluorescent-dye stained cells showed the highest reactive oxygen species (ROS) levels and mitochondrial membrane potential (Δψm) in cells with the highest lipid content, supporting the known relationship between mitochondrial activity and ROS generation. High intracellular ROS and low membrane integrity were not ameliorated by application of antioxidants.

**Conclusions:**

The limited intracellular energy supplies and the unbalanced redox environment could be regarded as targets for further lipid engineering, similarly for native lipid accumulation genes that were upregulated. Thus, lipid pathway engineering has an important effect on the central carbon pathway, directing these towards lipid production and sacrificing the precursors, energy and cofactor supply to satisfy homeostatic metabolic requirements.

**Electronic supplementary material:**

The online version of this article (10.1186/s13068-019-1435-6) contains supplementary material, which is available to authorized users.

## Background

Increasing market demand and broad applications of oleochemicals have made novel and sustainable routes to their production a target for research over the last decade [1]. Compared with the traditional production of plant oils and animal fats, the approach of using a microbial chassis for lipid production has the advantages of broad feedstock flexibility and availability [2]. Much progress in improving microbial lipid production has been made, bringing them closer to commercialization [3, 4]. Yeast, *Saccharomyces cerevisiae*, as a popular model and industrial microbe, has been widely investigated for lipid production and more broadly as a model for lipid engineering of oleaginous yeasts. Yeast engineered for enhanced standard and unusual lipids production have been constructed by a selective combination of genes supporting the steps of fatty acid biosynthesis and modification, lipid accumulation, and sequestration [5–7]. In our prior research, we observed that lipid pathway modification markedly increased the intracellular lipid content, but also led to reduced cell growth and membrane integrity which negatively affected the volumetric lipid yield [6].

Investigation of the basis of productivity loss and physiological impacts to cells subject to lipid pathway engineering may reveal mechanisms that could be addressed through further genetic modification or culture conditions, as examples. Two powerful approaches for revealing the responses of cells to metabolic engineering include transcriptome and physiological parameter analysis. While there have been few prior reports of transcriptomic analyses of yeast engineered for enhanced lipid production [8, 9], analysis to date has revealed likely oxidative stress impacts on cell growth and additional demand for NADPH in yeast engineered for high production of fatty acid esters. Technologies that have been used for transcriptome analysis include Sanger, microarray and RNA-seq, the latter shows advantages such as higher resolution, identification of novel transcripts and avoids background noise associated with fluorescence quantification and has become the dominant technology in research [10]. Regardless of the technology used, transcriptomics has been broadly applied to great effect in the study of cell metabolism [10].

Cell physiological measurements such as cell growth, soluble metabolite production, and lipid yields are commonly reported in yeast metabolic engineering research. However, these measures are limited by two aspects: they report an averaged response of the culture, and physiological states are inferred from metabolite profiles. Flow cytometry, when coupled with validated fluorescent dyes, can address these deficiencies as the technique measures cells individually and suitable dyes can be selected to measure intracellular functions. Flow cytometry has been widely used for the measurement of cell-membrane integrity (CMI) of cells treated with the fluorescent-dye propidium iodide, as a key measure of general cell health, and detection of reactive oxygen species (ROS) is often used to evaluate the cellular redox environment and oxidative stress. ROS is harmful to cells as it could damage DNA or RNA, oxidize polyunsaturated fatty acids, amino acids, and co-factors. Mitochondria are critical organelles implicated in the production of ROS in the cell as a normal consequence of aerobic metabolism [11] and their function can also be probed using flow cytometry with the addition of appropriate dyes. Mitochondrial membrane potential (Δψm) generated by proton pumps (Complexes I, III, and IV) is an important index for functional mitochondria [12].

**Fig. 1:**
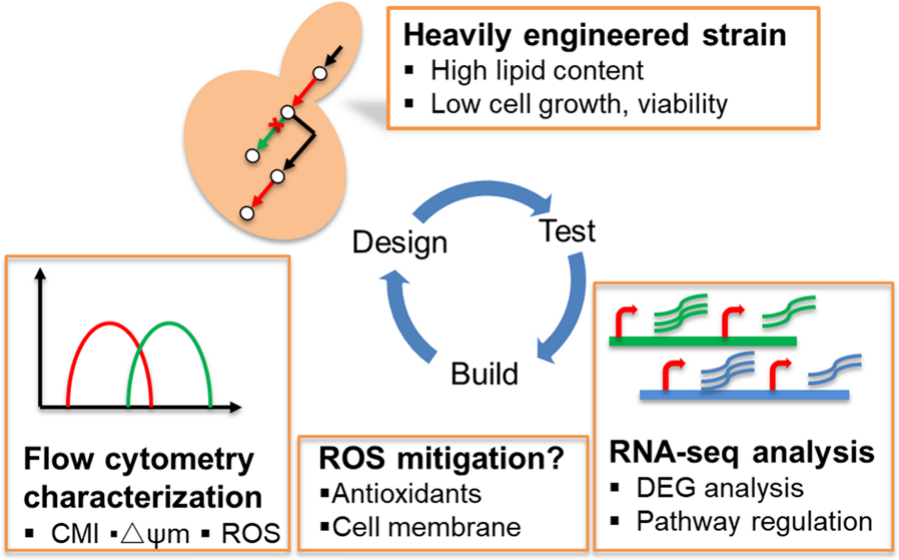
Schematic of the process flow for transcriptome and physiological response analyses of yeast cells engineered for enhanced lipid production. Heavily engineered yeast cells with high lipid content but lower cell growth and viability were first analysed by RNA-seq to identify the key DEG and pathway regulation, which were correlated with the physiological parameters of CMI, ROS, and Δψm, characterized by flow cytometry to determine future metabolic engineering targets. Oxidative stress mitigation was attempted by addition of antioxidants or cell-membrane modification. DEG, differentially expressed genes; CMI, cell-membrane integrity; ROS, reactive oxygen species; Δψm, mitochondrial membrane potential

Here, we employed complementary technologies of RNA-seq transcriptomic analysis and flow-cytometry-based physiological characterisation to investigate the performance and viability of yeast at different stages of lipid pathway engineering (Fig. 1). In this study, engineered strains of *S. cerevisiae* were compared by genome-wide transcription analysis and functional assessment of cell-membrane integrity and mitochondria and these were supported by selected metabolite analyses. The purpose of the study was to identify the impacts of lipid pathway engineering on cell physiology and potential further engineering targets for enhanced lipid production and improved cell health, towards the ultimate goal of improving production to economically competitive levels.

## Results and discussion

### Global transcriptional profiling emphasising central metabolic and lipid-related pathways

#### Whole transcriptome view of gene expression

Lipid pathway modification in yeast incorporated three steps including FA biosynthesis, lipid accumulation, and sequestration. H-series yeast strains including H03, 14, 20, 24, 25, 27, 28, and 31 were built as per the gene combinations, as shown in Table 1. Specifically, strains H03 and H14 were constructed by enhancing lipid accumulation and sequestration through heterologous expression of the diacylglycerol acyltransferase gene from *Arabidopsis thaliana* (*AtDGAT1*) on the parent strains BY4741 and BY4741 *TGL3*Δ, respectively. Strains H20, 24, and 25 were built on H14 by adding genes for FA biosynthesis *ALD6* (a native aldehyde dehydrogenase gene) and an acetyl-CoA synthetase variant from *Salmonella enterica* (*SeACS*^*L641P*^), or by increasing malonyl-CoA supply through the expression of ACC1** (a native acetyl-CoA carboxylase gene carrying two mutations ser659ala, and ser1157ala), or by the addition of caleosin, a lipid droplet-stabilising protein from *Arabidopsis thaliana* (*AtCLO1*). Strains H27, 28, and 31 were built on H20, by expressing ACC1**, or by expressing *AtCLO1*, or by co-expressing ACC1** and *AtCLO1*, respectively. All heterologous gene expressions were plasmid-based.

**Table 1:** Details of plasmids and strains used in this study

Plasmid or strain	Description	References
pYES2-Ura-*Ec.CFAS*	P_GAL1_-*Ec.CFAS*	[6]
pIYC05	P_PGK1_-*ALD6*/P_TEF1_-*SeACS*^*L641P*^, Addgene plasmid # 64742	[32]
pAD	P_PGK1_-ACC1** (ser659ala, ser1157ala), Addgene plasmid #64747	[32]
pESC-Leu-*AtDGAT1*	pESC-leu-P_GAL1_-*AtDGAT1*	[6]
pESC-Leu-*AtDGAT1*-*AtCLO1*	pESC-leu-P_GAL1_-*AtDGAT1*/P_GAL10_-*AtCLO1*	[6]
pESC-leu2d	Vector backbone, Addgene plasmid # 20120	[33]
pIYC04	Vector backbone, derived from pESC-His, Addgene plasmid # 64741	[32]
pSP-GM2	Vector backbone, derived from pESC-Ura, Addgene plasmid # 64740	[34]
Control	BY4741-pESC-leu2d-pIYC04-pSP-GM2	[6]
H03	BY4741–*AtDGAT1*	[6]
H14	BY4741Δ*TGL3*–*AtDGAT1*	[6]
H20	BY4741Δ*TGL3*–*AtDGAT1*–*ALD6*–*SeACS*^*L641P*^	[6]
H24	BY4741Δ*TGL3*–*AtDGAT1*–ACC1**	[6]
H25	BY4741Δ*TGL3*–*AtDGAT1*–*AtCLO1*	[6]
H27	BY4741Δ*TGL3*–*AtDGAT1*–ACC1**–*ALD6*–*SeACS*^*L641P*^	[6]
H28	BY4741Δ*TGL3*–*AtDGAT1*–*AtCLO1*–*ALD6*–*SeACS*^*L641P*^	[6]
H31	BY4741Δ*TGL3*–*AtDGAT1*–*AtCLO1*–ACC1**–*ALD6*–*SeACS*^*L641P*^	[6]
C20	BY4741Δ*TGL3*–*Ec.CFAS*–*AtDGAT1*–*ALD6*–*SeACS*^*L641P*^	[31]

**Fig. 2:**
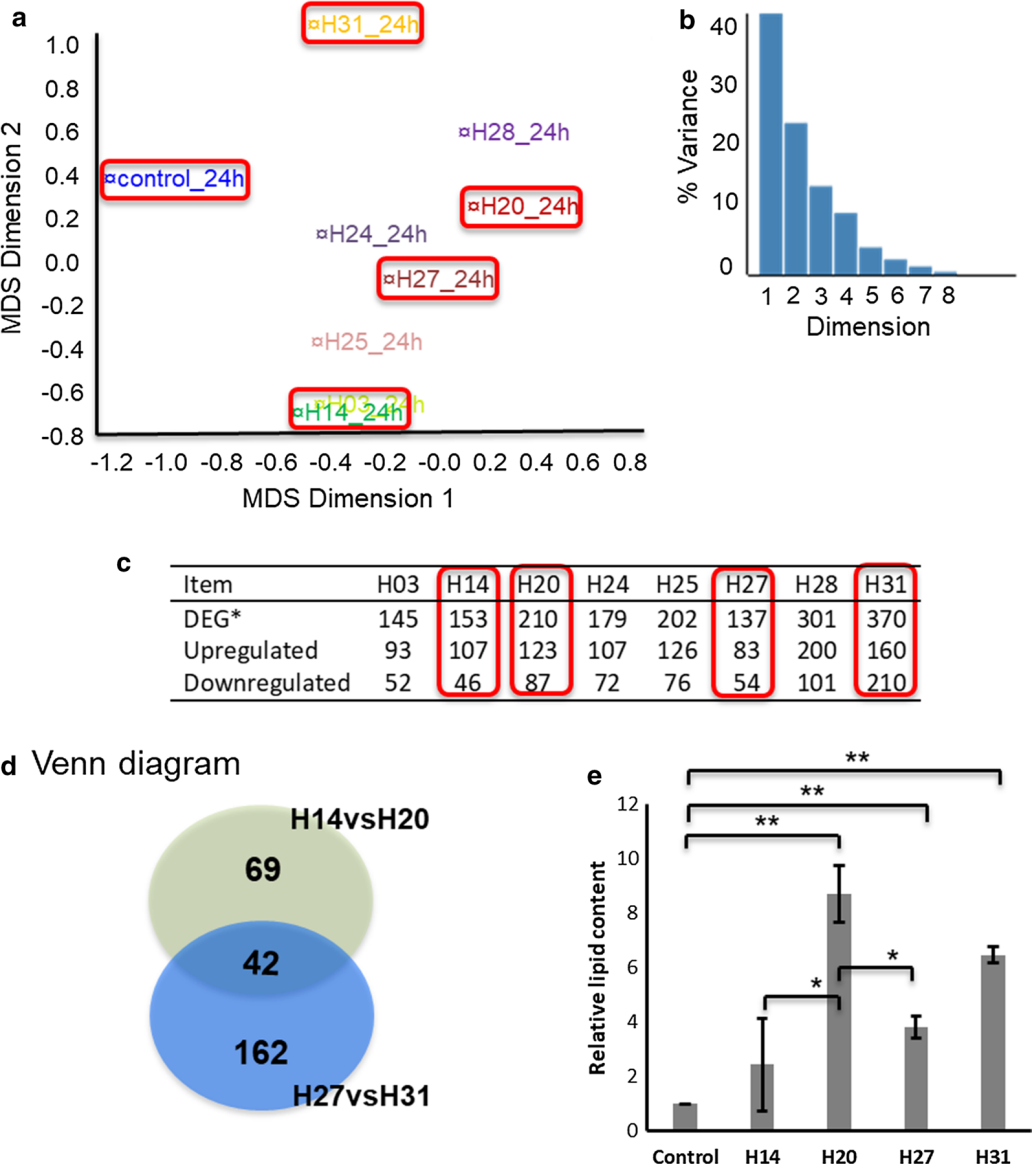
Overview of gene expression among yeast strains engineered for increased lipid production and cellular lipid content. **a** Multidimensional scaling (MDS) plot of gene expressions among different strains, **b** variance percentage by MDS of different dimensions. The MDS plot was generated by the Degust program, which was a means of visualizing the similar gene expression level of individual strain of the RNA-seq data set. The between-strain distance in the MDS plot determined the similarity of overall gene expression between two H-series strains. **c** Number of differentially expressed genes (DEG, including up- and downregulated) between engineered strains and control, **d** venn diagram of DEG between selected engineered strains H14, 20, 27, and 31. **e** Comparison of relative lipid content between four target strains using Nile red staining by flow cytometry, the lipid content of control was normalized to 1. *p < 0.05, **p < 0.01 (Student’s *t* test: two-tailed, two-sample equal variance)

The transcriptome-wide view of gene expression from RNA-seq analysis of engineered strains at 24 h post-induction in galactose media was analysed using the interactive web-tool Degust (http://degust.erc.monash.edu/) which has an element of principal component-like analysis developed specifically for transcriptomics data [13]. As shown in Fig. 2a, the multidimensional scaling (MDS) plot was generated by Degust, which is a means of visualizing the similar gene expression level of individual strains in the RNA-seq data set. The MDS algorithm placed each RNA-seq data set in *N*-dimensional space to preserve the between-strain distance, and each strain was then assigned a coordinate in each of the *N* dimensions. The between-strain distance in the MDS plot determines the similarity of overall gene expression between two H-series strains. The sum percentage of Dimensions 1 and 2 was > 60% (Fig. 2b), which indicated that these were the major components representing gene expression in the strains.

The numbers of differentially expressed genes (DEG) in the engineered strains versus control were compared and the data summarized in Fig. 2c, including the number of total DEG, upregulated genes and downregulated genes. Based on the number of total DEG, the 8 strains could be divided into three groups, one group with total DEG ~ 150 including strain H03, 14, and 27, one group with total DEG ~ 200 including strain H20, 24, and 25, another group with total DEG ~ 300 including strain H28 and 31. Taken together, the distance in the MDS plot and the DEG numbers resulted in four representative engineered strains H14, 20, 27, and 31 selected for detailed transcriptome analysis to explore relationships between DEG and lipid pathway modification. The Venn diagram (Fig. 2d) illustrates that there were 42 DEG among the four-engineered strains H14, 20, 27 and 31, and 111 DEG between H14 and H20, 204 DEG between H27 and H31. Of interest was that the heterologous expression of *ALD6*, *SeACS*^*L641P*^, and/or *AtCLO1* caused more DEG in strains H20, 28, and 31. As sequences for plasmid-expressed *ALD6* and minor mutant ACC1** were indistinguishable from the native counterparts in the RNA-seq analysis, their expression level was the sum of the overexpressed and native genes in the engineered strains but just native *ALD6* and *ACC1* transcripts in control yeast.

#### Expressions of target genes of interest support lipid-engineering strategy

Regarding the expression levels of introduced genes (Additional file 1: Fig. S1), *ALD6* was generally higher and ACC1** lower than other target genes compared to the control. Of further note, the ACC1** expression level was almost fivefold higher in H27 than other strains. H20 displayed higher *ALD6, AtDGAT1* expression levels than control and H14. Moreover, *AtDGAT1*, *AtCLO1* in H31 showed almost ninefold higher expression levels than other strains. Among the four-engineered strains, H20 and H31 showed higher lipid content at 24 h post-induction, followed by H27 and H14 and there was no significant difference in lipid level at 24 h post-induction between strains H20 and H31, as shown in Fig. 2e. Metabolite analyses of the culture media at this timepoint showed that there were sufficient sugars remaining to be considered the primary carbon sources for the yeast with ethanol and glycerol also present (Additional file 1: Fig. S2A–D). After 72 h, strain H31 showed significantly higher lipid content by dry cell weight (8.0%, DCW) compared with H20 (7.1%, DCW) [6]. The measured lipid levels of these strains supported the effectiveness of the engineering strategy.

The two engineered strains H20, 31 with higher lipid content at 24 h post-induction showed significantly reduced cell-membrane integrity (CMI), but strains H14 and 27 did not (described further below). This phenomenon suggested that further investigation of the relationship between reduced CMI, central metabolic pathway regulation and lipid production pathway modification was required.

#### Regulation of central carbon pathway and lipid production pathway

**Fig. 3:**
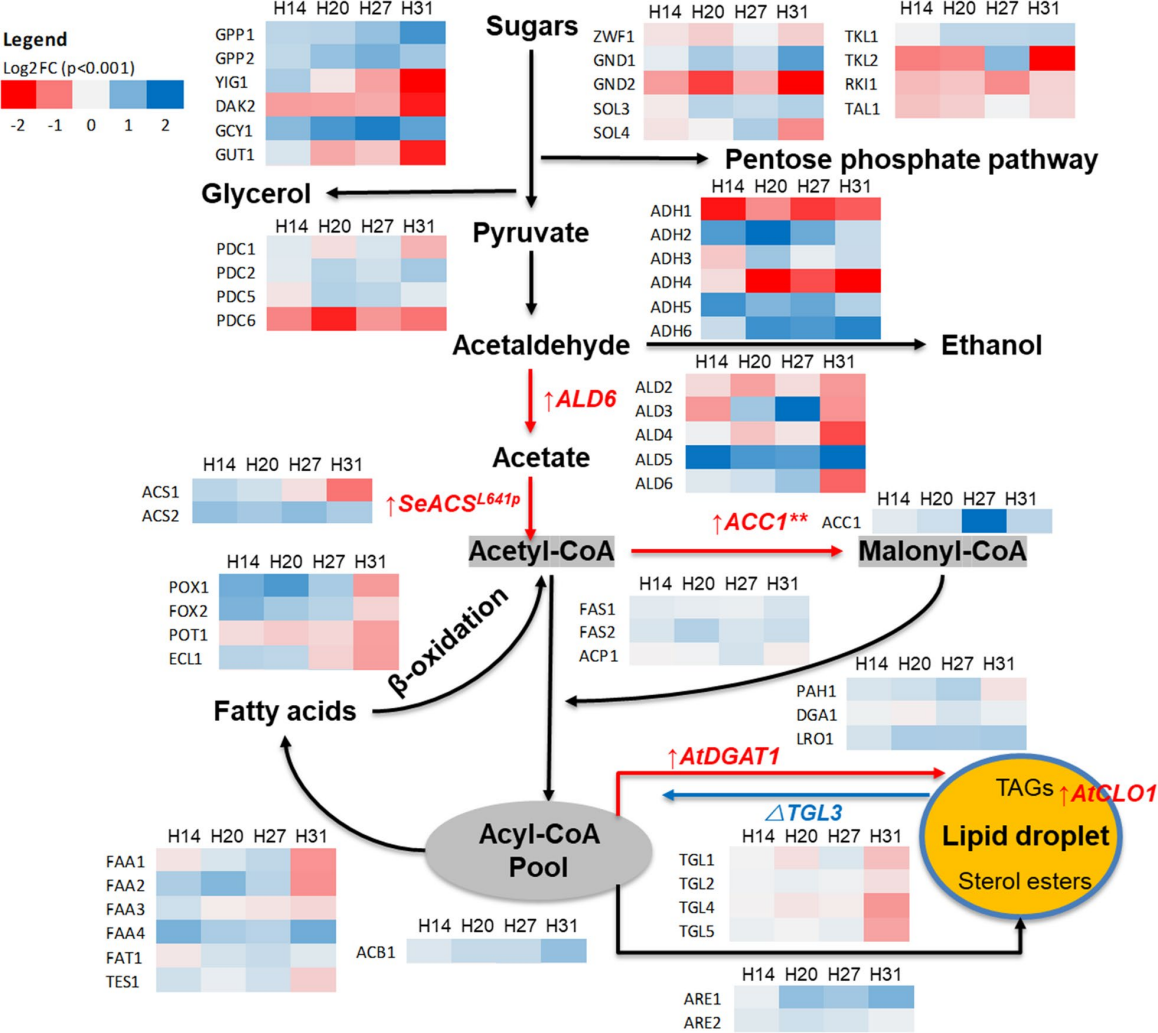
Overview of transcriptional reprogramming of the H-series strains H14, 20, 27, and 31 at 24 h post-induction of gene expression. The heatmaps show transcriptional differences in log2(fold change) (*p* < 0.001) in H-series strains compared with control strain, and mainly covered the central metabolic and lipid production pathway

The genes responsible for generating central metabolism precursors for fatty acid biosynthesis such as acetyl-CoA, malonyl-CoA, and acyl-CoA were upregulated in the target strains similar to the pattern of transcriptional rewiring within the central carbon pathway seen in *Y. lipolytica* engineered for enhanced lipid production via a different approach [14]. Expression profiles of orthologues of the introduced genes for FA biosynthesis such as *ALD5*, *ACS2*, and *ACC1* were upregulated (Fig. 3) which likely contributed to overall fatty acid synthesis. Furthermore, genes coding for fatty acid biosynthesis pathway-related enzymes were also upregulated including fatty acid synthetases (Fas1p, Fas2p) and acyl-CoA-binding protein (Acb1p). In strains H14, 20, and 27, acetyl-CoA synthetase (Acs1p) and fatty acyl-CoA synthetases (Faa1-4p, Fat1p) were upregulated to enhance fatty acid biosynthesis and activation to form fatty acyl-CoA, while the fatty acid consumption process via β-oxidation (*TES1*, *POX1*, *FOX2*) was also upregulated. Differing from the other three strains, H31 showed lower expression of several genes for FA biosynthesis (*ACS1*, *FAA1*-*3*, *TES1*) and β-oxidation (*POX1*, *FOX2*, *POT1*, *ECL1*). In its response to pathway engineering, H31 showed greater similarity to the transcriptome responses of a yeast strain expressing similar FA biosynthesis genes in an earlier report [8] than to H14, 20, and 27.

In the lipid accumulation and sequestration pathways, heterologous expression of *AtDGAT1* did not change the transcription level of the endogenous *DGA1*, except a slight upregulation in H27 (Fig. 3); similarly, the knockout of *TGL3* did not obviously change the expression of other TAG lipase genes *TGL1*-*2* and *TGL4*-*5* in the engineered strains except for the downregulation of these in H31 (Fig. 3). Interestingly, *PAH1*, *LRO1,* and *ARE1*,*2*, genes responsible for diacylglycerol/lipid droplet, triglyceride, and sterol synthesis, respectively, were upregulated in the four target strains, which suggests that our current lipid accumulation and sequestration strategy was insufficient for demand and additional FA sequestration, as lipid or sterol sterols was required. Thus, *PAH1*, *LRO1,* and *ARE1,2* are interesting targets for future lipid engineering research. *ARE1* and *LRO1* were more highly upregulated in H31 and TAG lipases were more downregulated compared with strain H27; these two strains differ only by the addition of *AtCLO1*, a lipid droplet associated protein from *A. thaliana* [15]. The pattern of upregulation suggested that there was a higher demand in H31 for FA accumulation enzymes. Caleosin was proposed to modify lipid droplet membrane function and increase the size of droplets by impairing lipase accessibility to lipid bodies, slowing storage lipid degradation [15]. This function is due to the special non-canonical structure of caleosin, with a long central hydrophobic region surrounded by two hydrophilic C- and N-terminal regions [16].

### Regulation of redox pathway and cellular physiological characterisation

#### Effect of lipid pathway engineering on redox pathways

The pentose phosphate pathway (PPP) is considered to be the primary source of NADPH supply in yeast [17] which is highly consumed during the reduction steps of fatty acid synthesis. The engineered strains showed that overall downregulation of key genes involved in the PPP including *ZWF1*, *GND2,* and *RKI1* which suggests that NADPH supply was impaired in these strains (Fig. 3). *GND2* expression responses were also strongly downregulated in yeast cells engineered for fatty ester production [8]. Interesting, gene *TKL2*, encoding transketolase (a key enzyme in the PPP pathway), was upregulated in strain H27 while downregulated in H14, 20, and 31. This could be explained by the fact that much higher expression levels of *ALD6* and *ACC1* in H27 were supported by a better NADPH supply in H27 than other target strains.

Compared to control yeast, the glycerol production pathway encoded by *GDP1, GDP2, GPP1,* and *GPP2* was upregulated and the glycerol catabolism pathway encoded by *YIG1, DAK2,* and *GUT1* was downregulated in the engineered strains (Fig. 3). This agreed with higher media glycerol concentrations in these strains compared with the control (Additional file 1: Fig. S2D). An upregulated glycerol biosynthesis pathway may have been required to balance NADH and osmotic stress [18]. Gene expression for the ethanol biosynthesis pathway, e.g., alcohol dehydrogenase (*ADH1*) was downregulated in engineered strains together with upregulation of ethanol catabolism genes (e.g., *ADH2*); this result agreed with lower ethanol production in engineered strains such as H14 and 20 (Additional file 1: Fig. S2C). Strain H27 showed comparable ethanol production with control yeast and higher than H14 and 20, which was likely due to the upregulated alcohol dehydrogenase genes (*ADH5*, *ADH6*). Furthermore, the upregulation of *TKL2* (encoding transketolase, a key enzyme in the PPP pathway) in strain H27 would generate higher NADPH production to support ethanol production (Fig. 3).

Overall, lipid pathway engineering impaired redox pathways and redirected carbon flux from ethanol biosynthesis to lipid and glycerol production. This was similar to the effect of *MHY1* gene inactivation in *Y. lipolytica*, a crucial gene in lipid production regulation, which increased carbon flux to lipogenesis from amino acid biosynthesis [19]. As lipid biosynthesis and derived products require a large quantity of NADPH (synthesis of one palmitate requires 14 NADPH), increasing NADPH supply has been the focus of several studies to improve the production of ethanol and fatty esters [20, 21]. Thus, downregulation of the PPP and β-oxidation may explain the slow growth of the more engineered strains H20 and H31 and reinforce the importance of balancing cofactor supply in lipid pathway engineering research.

#### Effect of lipid pathway engineering on ROS production and mitochondrial function

**Fig. 4:**
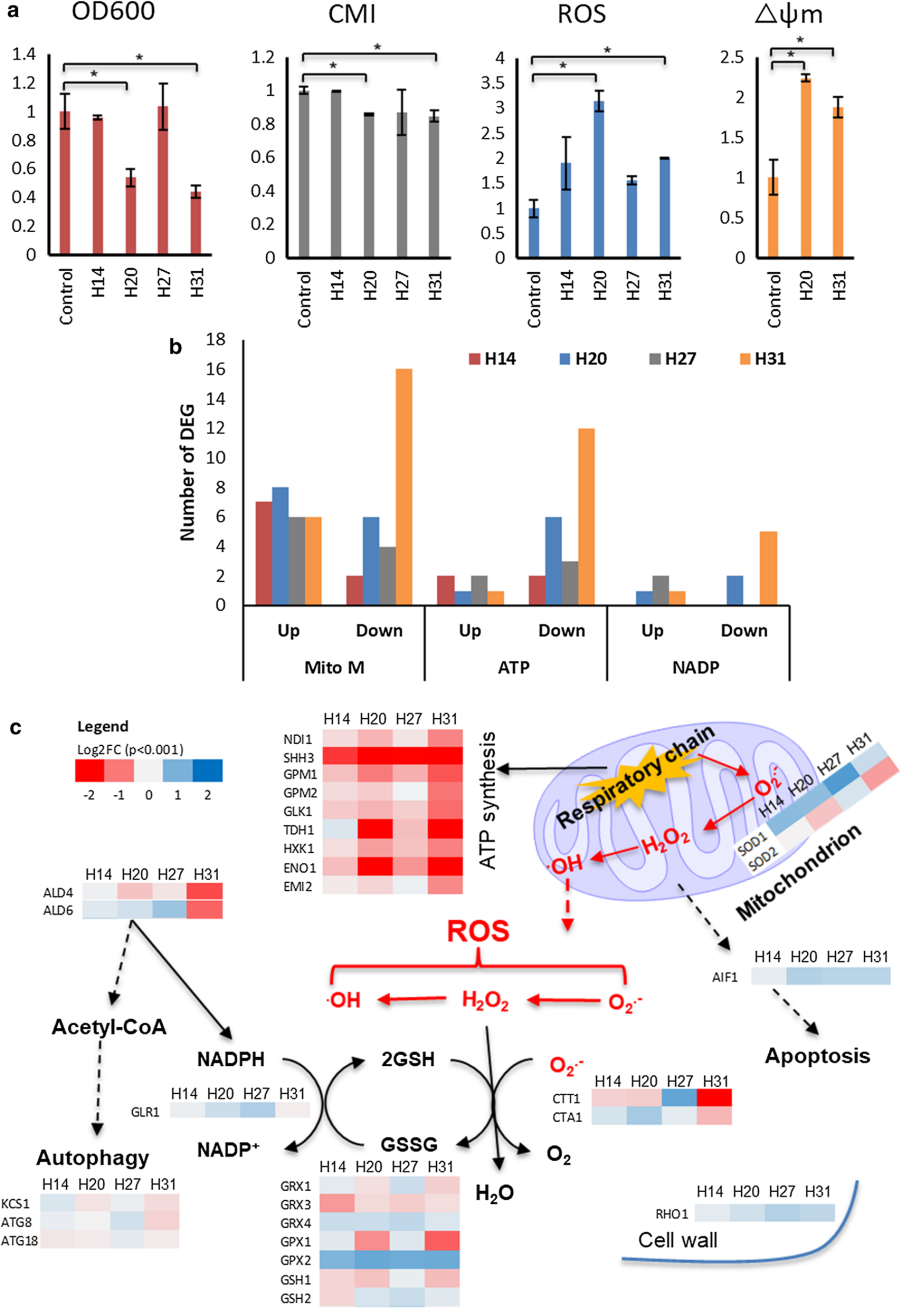
Cellular physiological characterisation and related differentially expressed genes in strains H14, 20, 27, and 31 compared with control 24 h post-induction of gene expression: **a** comparison of cell growth by OD_600_ nm, CMI (cell-membrane integrity), intracellular ROS (reactive oxygen species), and Δψm (mitochondrial membrane potential), **b** number of differentially expressed genes as per gene ontology including Mito M (mitochondrial membrane, GO: 0031966), ATP (ATP metabolic process, GO: 0046034), and NADP (NADP metabolic process, GO: 0006739). **c** Differentially expressed genes in the ROS production-related pathways and mitochondria between H-series strains and control. The heatmaps showed the transcriptional differences in log2(fold change) (*p* < 0.001). *p < 0.05 (Student’s *t* test: two-tailed, two-sample equal variance)

Cell growth and CMI were much lower for strains H20 and 31, accompanied by higher intracellular ROS compared with the other strains (Fig. 4a) and with comparable lipid content at 24 h. We speculated that lower CMI may be due to high ROS level in the cell [22] and that this could arise from mitochondrial respiration or β-oxidation, or from impaired antioxidant defense systems. Since β-oxidation (*POX1, FOX2,* and *TES1*) was downregulated in strain H31 (Fig. 3), we reasoned that the other two sources were more likely to explain the high intracellular ROS value in this strain. However, while there was higher mitochondrial membrane potential (Δφm) measured in the H20 and 31 cells (Fig. 4a), more genes coding for mitochondrial enzymes and mitochondrial membrane synthesis were downregulated in these strains compared with control and H14 and 27 (Fig. 4b). The apparent contradiction between physiological and transcriptomic results may reflect dynamic changes occurring at the specific timepoint of sampling (24 h) and warrants further investigation over a broader timeframe. Meanwhile, the ROS level in strains H20 and 31 may be also a factor in the upregulation of the small G-protein Rho1p, which controls the cell-wall integrity pathway in response to oxidative stress [23].

ROS species including hydrogen peroxide (H_2_O_2_), superoxide radical (O_2_^−^), and hydroxyl radical (OH·) cause damage to cells and are metabolised via multiple pathways (indicated in Fig. 4c). In H20 and 31, genes including *TDH1* and *ENO1* involved in the respiratory chain and ATP synthesis were downregulated and in the conversion of H_2_O_2_ from O_2_^**−**^ may have been affected by *SOD2* (encoding superoxide dismutase) downregulation. Furthermore, genes coding for catalase (*CTT1*), glutathione-dependent enzymes (*GPX1*, *GRX1*), and glutathione transferases (*GSH1*) which protect the cell from ROS damage were all downregulated in H20 and 31, but expression was either unchanged in H14 and 27 or showed the opposite. This loss of protection may explain the higher ROS level measured in H20 and 31 compared with control (Fig. 4a); however, the reason for downregulation of these genes is not clear.

Most genes responsible for ATP and NADPH synthesis were downregulated in the respiratory chain of strain H31 which indicated that the energy storage forms were unbalanced and reducing agent or cofactor were limited in these cells. As examples, Ndi1p, the internal mitochondrial NADH dehydrogenase that transfers electrons to complex III of the respiratory chain; Shh3p, a putative mitochondrial inner membrane protein; and two aldehyde dehydrogenases (*ALD4*, *6*) that contribute to NADPH synthesis, were downregulated. Besides, the autophagy-related genes including *KCS1*, *ATG8*, and *ATG18* were only mildly downregulated and the apoptosis-inducing factor gene (*AIF1*) was only mildly upregulated indicated that the slow cell growth of highly engineered strain H31 was not caused by large-scale upregulation of autophagy or apoptosis process. Therefore, unbalanced energy and reducing equivalents supply in strain H31 and by extension to strain H20, could lower cell growth and reduce CMI.

### Potential for oxidative stress generation in lipid pathway engineering

#### Effect of antioxidants

**Fig. 5:**
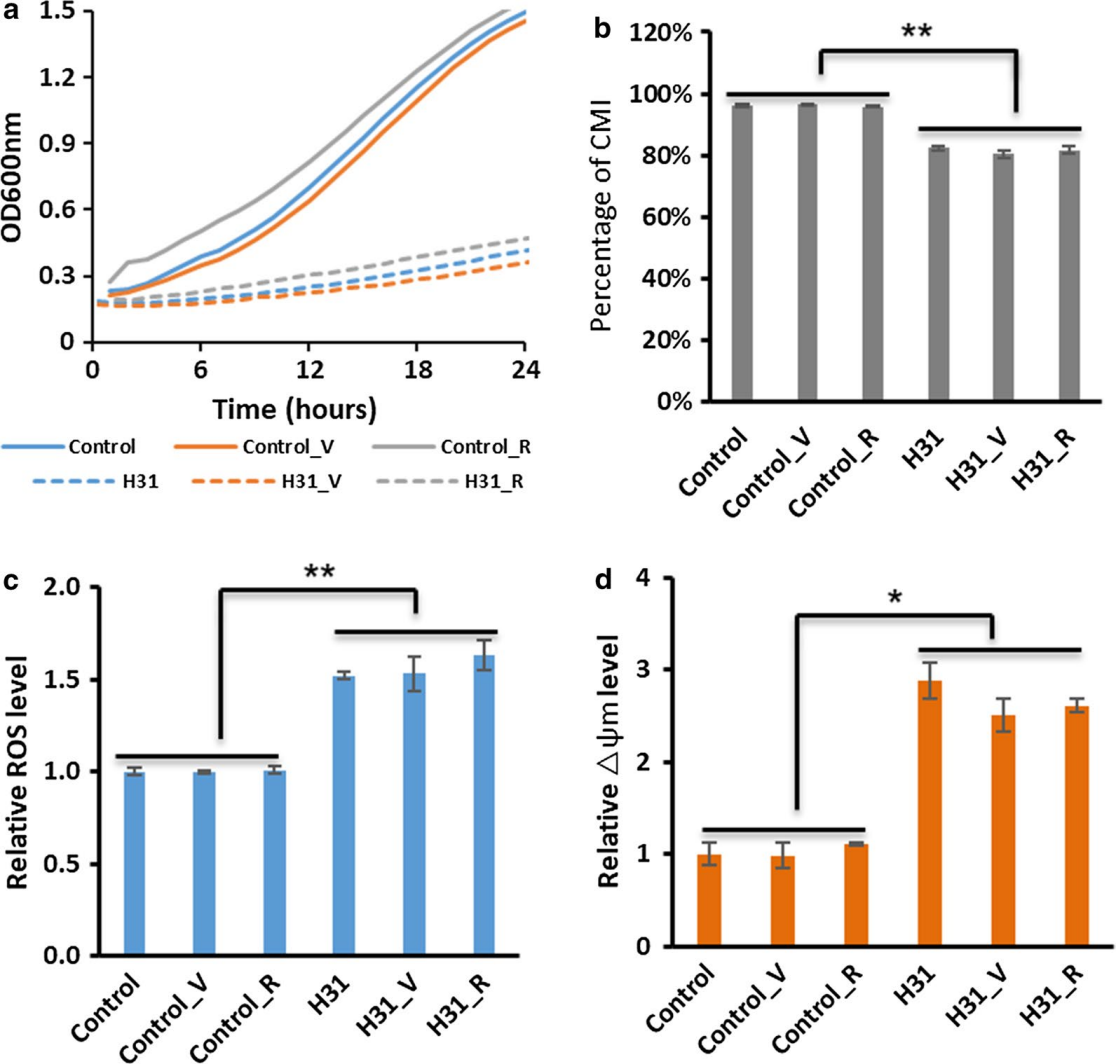
Comparison of cellular physiological responses between strain H31 and control after addition to media of antioxidants Vitamin C (_V) and resveratrol, (_R). **a** Cell growth of control and H31 strains over 24 h in 48-well plate with stirring, **b** cell-membrane integrity (CMI), **c** relative reactive oxygen species level (ROS) normalized to 1 for control and **d** relative mitochondrial membrane potential (Δψm) at 24 h post-induction normalized to 1 for control. *p < 0.05, **p < 0.01 (Student’s *t* test: two-tailed, two-sample equal variance)

Engineering oxidative stress defense pathways in yeast alleviated ROS and improved standard lipid and PUFA production in the previous studies [3, 4, 24]. In addition, Ma et al. 2019 succeeded in combining the engineering of lipid and lycopene (antioxidant) synthesis pathways to markedly increase production of both products using a two-stage bioprocess [25]. In our experiments, strain H31 consistently showed elevated intracellular ROS levels (Fig. 4a); thus, we proposed to ameliorate this by the addition of antioxidants to the culture medium. Two well-studied antioxidants, vitamin C and resveratrol, had previously demonstrated the ability to moderate intracellular ROS level in yeast [26, 27], and these were applied, separately, to yeast expression induction medium of control and strain H31 and the effects were monitored for 24 h. Contrary to our expectations, the antioxidants vitamin C and resveratrol did not have a significant effect on any of the physiological parameters measured in the control or H31 strains. That is, neither growth, CMI or intracellular content of ROS significantly improved in strain H31 by application of antioxidants (Fig. 5); mitochondrial membrane potential was similarly unaffected. This result showed that while strain H31 has relatively high ROS levels and low CMI, these cannot be ameliorated by the addition of classical antioxidants.

#### Cell-membrane modification by cyclopropane fatty acid biosynthesis

**Fig. 6:**
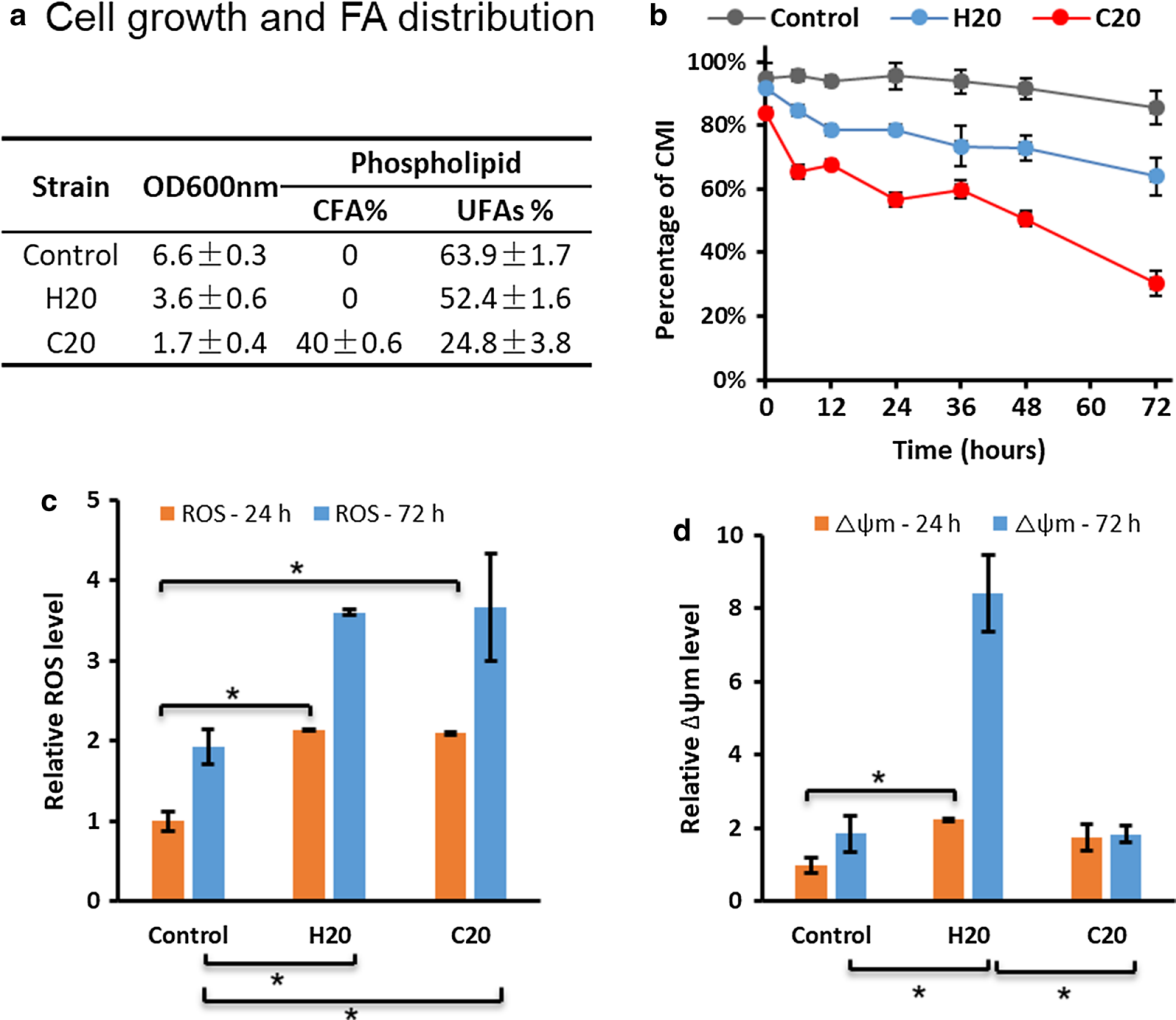
Effect of cyclopropane fatty acid presence in cell membranes on the cellular physiological performance of yeast strain C20 compared with control and H20 which lack the unusual fatty acid. **a** Comparison of cell growth by OD_600_ nm and fatty acid profile of the cell-membrane phospholipid at 72 h post-induction; UFAs, unsaturated fatty acids; CFAs, cyclopropane fatty acids, **b** comparison of time course of percentage of CMI (cell-membrane integrity), **c** relative ROS level at 24 h and 72 h, the ROS level of control at 24 h was normalized to 1, **d** relative Δψm level at 24 h and 72 h, the Δψm level of control at 24 h was normalized to 1. *p < 0.05 (Student’s *t* test: two-tailed, two-sample equal variance)

Cyclopropane fatty acid (CFA) incorporation into cellular phospholipid membranes has been reported to increase tolerance in microorganisms such as *E. coli* in response to an acidic environment [28] and to more generalized stresses. The higher oxidative stability of CFA compared with unsaturated fatty acids may also be an advantage in the protection of membranes from ROS attack which targets (poly)unsaturated fatty acids in the first instance. We hypothesised that CFA in membranes might improve membrane integrity and hence cell growth in compromised engineered yeast strains. To test this hypothesis, two strains were directly compared: H20 expressing *AtDGAT1*, *ALD6*-*SeACS*^*L641P*^, Δ*TGL3*, and C20 which contained the same heterologous genes plus an additional cyclopropyl fatty acid synthesis gene from *E. coli* (*Ec.CFAS*). CFA concentration in the phospholipid fraction of C20 was measured at ~ 40% and unsaturated FA percentage was reduced from 64 to 25% (Fig. 6a). This result verified the inclusion of CFA into phospholipid membranes in the C20 strain.

Cell growth of C20 was lower (Fig. 6b), and ROS levels were unchanged from that for H20 (Fig. 6c), whereas mitochondrial membrane potential (Δψm) was similar between C20 and control and did not show the large increase that H20 exhibited at 72 h (Fig. 6d). These outcomes could be explained by the requirement for unsaturated FAs in maintaining membrane fluidity and cell growth in *S. cerevisiae* which is not satisfied by incorporation of CFA [29, 30] and that ROS generated internally may not be targeting membrane phospholipids and thus not damaging membrane integrity.

## Conclusions

This study investigated the relationships between transcriptomic profile and cellular physiological responses of yeast engineered for increased lipid production. The transcriptomic analysis showed that the lipid engineering strategy redirected the global metabolic pathway towards lipid production, such as upregulated pathways for the production of precursors including acetyl-CoA, malonyl-CoA, and acyl-CoA. Glycerol biosynthesis pathway was also upregulated and ethanol biosynthesis and the PPP were downregulated. ROS levels were consistently high in engineered strains but so was mitochondrial membrane potential; as mitochondria are the main source of ROS in the cell, high ROS is likely a result of the high mitochondrial activity. The high ROS and low membrane integrity of the engineered strain H31 were not reversed by the addition of antioxidants to the culture media or by altering the membrane lipids to include high concentrations of saturated CFA. These results suggest that current antioxidants treatment and membrane modifications are not efficient enough to mitigate elevated ROS levels or benefit membrane integrity and low cell growth. Moreover, the downregulated ATP and NADPH synthesis pathways suggested that the intracellular energy supply was limited and redox levels were unbalanced in the engineered cells where lipid accumulation pathway genes were upregulated. These are all promising targets for further lipid pathway engineering. Finally, the study shows the power of transcriptome and cell physiological analyses to examine cellular performance and direct future engineering efforts.

## Methods

### Strains

In this study, strains from previous research [6, 31] that were engineered for enhanced standard lipids and cyclopropane fatty acid production were selected for analysis. All the engineered strains were constructed based on the parent strain BY4741 (MATa his3Δ1 leu2Δ0 met15Δ0 ura3Δ0), and the details of plasmids and strains are listed in Table 1.

### Cell culture

Detailed culture conditions have been described earlier [6] and given briefly below. The engineered yeast strains were maintained based on their auxotrophy using yeast synthetic complete (SC) minimal medium, which contains 6.7 g/L of yeast nitrogen base, 20 g/L glucose plus a mixture containing appropriate nucleotide bases and amino acids for the various dropout options (SC-Leu, SC-His-Leu, and SC-His-Leu-Ura). For the induction medium, glucose was replaced with 2% (w/v) galactose and 1% (w/v) raffinose. The seed culture was prepared by inoculating a single transformed colony into 5 mL yeast SC minimal medium and incubating at 30 °C, 250 rpm, overnight. The culture was diluted using SC induction medium to an initial OD_600_ nm value of approximately 0.4 and then incubated at 30 °C and 250 rpm.

### Antioxidant addition to cell culture

Two antioxidants (vitamin C and resveratrol) were selected to assess their effects on the cell physiology of strain H31 during culturing. Cell growth was evaluated in the absence and the presence of vitamin C or resveratrol (0.5 μM and 2.5 mM, respectively) added to media in 48-well plates. The selected concentrations of vitamin C and resveratrol were based on our preliminary screening experiments and other previous reports [26, 35]. Seed cultures of control and H31 cells were dispensed into each well and diluted to an initial OD_600_ nm of 0.2. The microplates were incubated at 30 °C, and growth (OD_600_ nm) was measured every 30 min using the Tecan microplate reader [36]. Cell samples were taken at 24 h for flow-cytometry analysis.

### RNA-seq analysis

Samples for transcriptome analysis were taken at 24 h post-induction during late exponential phase growth. Approximately 20 mL cell culture for each strain was cooled directly to 4 °C and centrifuged to obtain the cell pellets, which were washed quickly with 0.2 μm filtered PBS buffer once and then centrifuged again. The cell pellets were directly frozen in liquid nitrogen and stored at − 80 °C before shipping for RNA-Seq analysis stored on dry ice. The RNA extraction, library preparation (250–300 bp insert cDNA library), RNA sequence analysis, and assembly were conducted by Novogene, Hong Kong. The detailed method is provided in Additional File 1.

### Cell physiology measurement

Cell growth was monitored by optical density (OD) at 600 nm using DR 5000™ UV–Vis spectrophotometer. Harvested cells were adjusted into 10^6^/mL (OD ~ 0.2), washed twice using fresh PBS buffer (pH 7.4), and resuspended in 1 mL PBS buffer for flow-cytometry analysis for neutral lipid content, cell-membrane integrity (CMI), reactive oxygen species (ROS) level, and mitochondrial membrane potential (Δψm). In the following analyses, stained cells were kept in the dark to avoid photobleaching. Individual cell fluorescence was measured using the violet laser (488 nm) on a CytoFlex (Beckman Coulter, USA) under PE or FITC filter. More than 10,000 cells were measured for each sample and the data acquired were analysed further by the software CytExpert Ver.2.0.

For lipid content analysis, Nile red was used to stain the cells at a final conc. of 5 μg/mL, and incubated at room temperature for 5 min before reading under PE filter. For CMI and ROS analyses, the cell samples were first stained with CM-H_2_DCFDA (Thermo Fisher Scientific, USA) at a final conc. of 5 μM and incubated at 30 °C, 250 rpm for 30 min, then stained with propidium iodine (PI) (ThermoFisher, USA) at a final conc. of 5 μg/mL, and analysed immediately by flow cytometry under both FITC and PE filter, for ROS level and CMI, respectively. TMRE (tetramethylrhodamine, ethyl ester) (Abcam, UK) was used to analyse the mitochondrial membrane potential (Δψm). Freshly washed cells were treated with TMRE at a final conc. of 100 nM, and incubated at 30 °C, 250 rpm for 20 min before flow-cytometry analysis using the PE filter. The depolarization (negative) control was prepared by treating the cells with 20 μM FCCP (carbonyl cyanide 4-(trifluoromethoxy) phenylhydrazone) 10 min before adding TMRE.

## Additional file

**Additional file 1.** Flow-cytometry-based physiological characterisation and transcriptome analyses reveal a mechanism for reduced cell viability in yeast engineered for increased lipid content.

## Abbreviations

PL: phospholipid
DCW: dry cell weight
TAG: triacylglycerol
CFA: cyclopropane fatty acid
FA: fatty acid
*ALD6*: overexpression of native aldehyde dehydrogenase isoform 6
*SeACS*^*L641P*^: acetyl-CoA synthetase with an L641P mutation from *S. enterica*
*AtDGAT1*: diacylglycerol acyltransferase from *A. thaliana*
*Ec.CFAS*: cyclopropane fatty acid synthase from *E. coli*
Δ*TGL3*: knock-out gene triglyceride lipase isoform 3
CMI: cell-membrane integrity
ROS: reactive oxygen species
Δψm: mitochondrial membrane potential
