# Supplementary material for: Flow-cytometry-based physiological characterisation and transcriptome analyses reveal a mechanism for reduced cell viability in yeast engineered for increased lipid content
Source: Biotechnol Biofuels. 2019 Apr 23;12:98. doi: 10.1186/s13068-019-1435-6 (PMC6477733; doi:10.1186/s13068-019-1435-6)
Supplement: Supplementary file 1 — Additional file 1. Flow-cytometry-based physiological characterisation and transcriptome analyses reveal a mechanism for reduced cell viability in yeast engineered for increased lipid content. [file 13068_2019_1435_MOESM1_ESM.docx]

For submission to: Biotechnology for Biofuels

Section: Research Paper

**Additional files for**

**Flow cytometry-based physiological characterisation and transcriptome analyses reveal a mechanism for reduced cell viability in yeast engineered for increased lipid content**

**Huadong Peng^1, 2^, Lizhong He^1^, Victoria S. Haritos^1,^ ***

^1^Department of Chemical Engineering, Monash University, Clayton, VIC 3800, Australia

^2^Present address: Department of Bioengineering, Imperial College London, London SW7 2AZ, UK

Email addresses:

[hdpeng89@gmail.com](mailto:hdpeng89@gmail.com); [lizhong.he@monash.edu](mailto:lizhong.he@monash.edu); [victoria.haritos@monash.edu](mailto:victoria.haritos@monash.edu)

Author for correspondence

Assoc Prof Victoria Haritos,

Department of Chemical Engineering,

Monash University, Clayton 3800, Victoria Australia

Phone: +61 3 9905 6873

Email: victoria.haritos@monash.edu


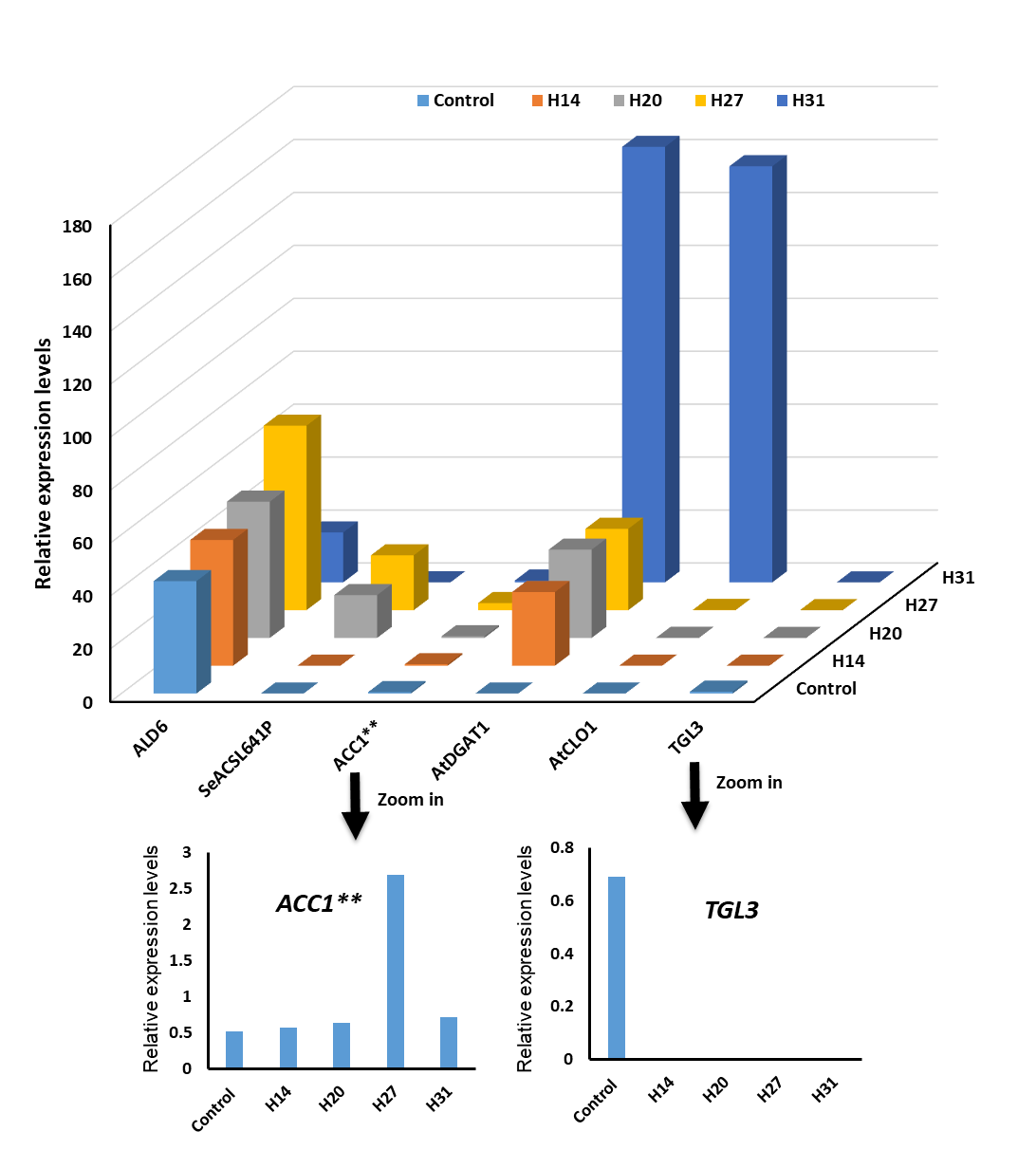


**Additional Figure S1** Relative expression levels of heterologously expressed genes among engineered strains by mRNA-seq analysis including *ALD6*, *SEACS^L641p^*, *ACC1***, *AtCLO1*, *TGL3* and *AtDGAT1*. Yeast strains were sampled for mRNA-Seq analysis at 24 h post-induction including Control, H14, 20, 27, 31. The gene expression levels of *ACC1*** and *TGL3* were enhanced separately due to their low levels. Expression levels of genes of interest were normalized against the expression level of *TAF10*, a gene that encodes a subunit of transcription factor IID (TFIID) and regarded as a housekeeping gene.


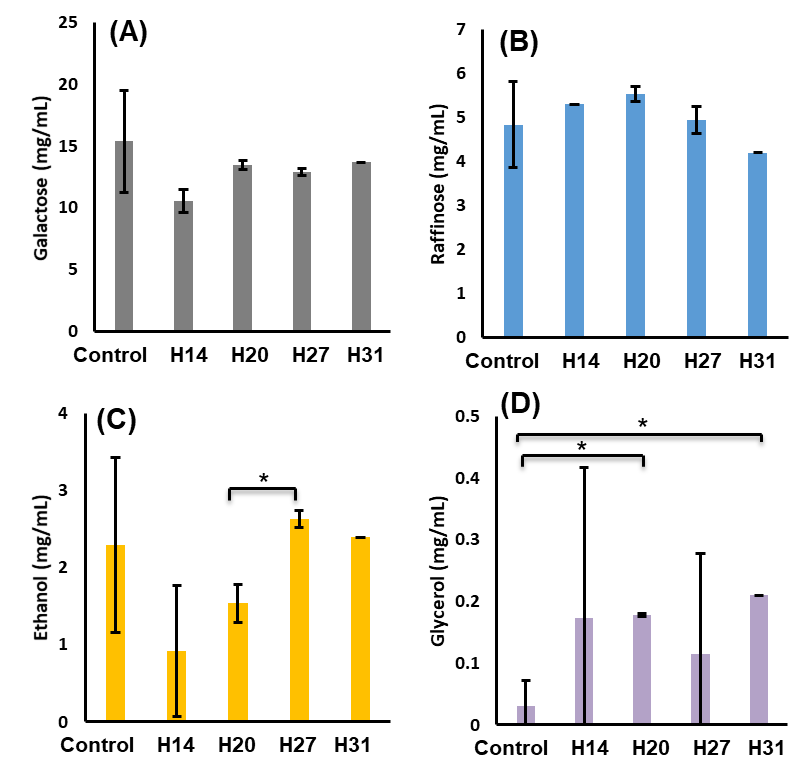


**Additional file Figure S2**

Sugars and soluble metabolite concentrations remaining in supernatants of yeast strains at the 24 h timepoint post-induction. (A) Galactose concentration, mg/mL, (B) Raffinose concentration, mg/mL, (C) Ethanol concentration, mg/mL, (D) Glycerol concentration, mg/mL. *P < 0.05. (Student's t-test: two-tailed, two-sample equal variance).

## Methods

### RNA-seq analytical method

The following RNA-seq analytical method has been reproduced from Novogene company and Zhao et al. 2015 [1] with slight modifications.

**RNA isolation, examination and qualification**

RNeasy Mini Kit (QIAGEN, Alameda, USA) was used to isolate and purify the total RNA as per the production protocol. 1% agarose gels and NanoPhotometer® spectrophotometer (IMPLEN, CA, USA) were used to check the RNA degradation, contamination and purity, respectively. Besides, RNA concentration and integrity were assessed by Qubit® RNA Assay Kit in Qubit® 2.0 Flurometer (Life Technologies, CA, USA) and RNA Nano 6000 Assay Kit of the Bioanalyzer 2100 system (Agilent Technologies, CA, USA), respectively.

**Library preparation for Transcriptome sequencing**

A total amount of 3 mg RNA of each sample was used for the RNA sample preparations. Sequencing libraries were generated using NEBNext® Ultra™ RNA Library Prep Kit for Illumina® (NEB, USA) following manufacturer's instruction and index codes were added to attribute sequences to each sample. Briefly, mRNA was purified from total RNA using poly-T oligo-attached magnetic beads. Fragmentation was carried out using divalent cations under elevated temperature in NEBNext First-Strand Synthesis Reaction Buffer (5×). First strand cDNA was synthesized using random hexamer primer and M-MuLV Reverse Transcriptase (RNase H^-^). Second strand cDNA synthesis was subsequently performed using DNA polymerase I and RNase H. Remaining overhangs were converted into blunt ends via exonuclease/polymerase activities. After adenylation of 3'ends of DNA fragments, NEBNext Adaptor with hairpin loop structure were ligated to prepare for hybridization. In order to select cDNA fragments of preferentially 150–200 bp in length, the library fragments were purified with AMPure XP system (Beckman Coulter, Beverly, USA). Then 3 mL USER Enzyme (NEB, USA) was used with size-selected, adaptor-ligated cDNA at 37 ℃ for 15 min followed by 5 min at 95 ℃ before PCR. Then PCR was performed with Phusion High-Fidelity DNA polymerase, Universal PCR primers and Index (X) Primer. At last, PCR products were purified (AMPure XP system) and library quality was assessed on the Agilent Bioanalyzer 2100 system (Agilent Technologies, Palo Alto, USA). The clustering of the index-coded samples was performed on a cBot Cluster Generation System using TruSeq PE Cluster Kit v3-cBot-HS (Illumina Inc., San Diego, CA, USA) according to the manufacturer's instructions. After cluster generation, the library preparations were sequenced on an Illumina Hiseq 2000 platform and 50 bp single-end reads were generated.

**Transcriptome data analysis**

All analyses were based on clean data with high quality reads after removing those that contained adapter, poly-N or low quality reads from the raw data. For the reads mapping, the reference genome and gene model annotation files of *S. cerevisiae* were downloaded from the *Saccharomyces* Genome Database (SGD). Index of the reference genome was built using Bowtie v2.2.3 and paired-end clean reads were aligned to the reference genome using TopHat v2.0.12. RPKM referred to the reads per kilobase of exon model per million mapped reads. Prior to differential gene expression analysis, HTSeq v0.6.1 was used to count the reads numbers mapped to each gene. And then the RPKM of each gene was calculated based on the length of the gene and reads count mapped to this gene [2]. For each sequenced library, the read counts were adjusted using the edgeR software package through one scaling normalized factor [3]. Differential expression analysis of two conditions was performed using the DESeq R package (1.20.0) as described by Anders and Huber [4]. The p values were adjusted using the Benjamini & Hochberg method. Corrected p-value of 0.005 and log2(Fold change) of 1 were set as the threshold for significantly differential expression. Gene ontology (GO) was implemented using the GOseq R package, in which gene length bias was adjusted [5]. GO terms with corrected p-value less than 0.05 were considered significantly enriched by differential expressed genes. In order to examine the associated pathways and functions in the differential expressed genes, KOBAS software 2.0 was employed in KEGG pathway mapping analysis [6].

**References**

1. Zhao H, Chen J, Liu J, Han B: **Transcriptome analysis reveals the oxidative stress response in Saccharomyces cerevisiae**. *Rsc Adv* 2015, **5**(29):22923-22934.

2. Mortazavi A, Williams BA, McCue K, Schaeffer L, Wold B: **Mapping and quantifying mammalian transcriptomes by RNA-Seq**. *Nature methods* 2008, **5**(7):621.

3. Robinson MD, McCarthy DJ, Smyth GK: **edgeR: a Bioconductor package for differential expression analysis of digital gene expression data**. *Bioinformatics (Oxford, England)* 2010, **26**(1):139-140.

4. Anders S, Huber W: **Differential expression analysis for sequence count data**. *Genome biology* 2010, **11**(10):R106.

5. Trapnell C, Williams BA, Pertea G, Mortazavi A, Kwan G, Van Baren MJ, Salzberg SL, Wold BJ, Pachter L: **Transcript assembly and quantification by RNA-Seq reveals unannotated transcripts and isoform switching during cell differentiation**. *Nature biotechnology* 2010, **28**(5):511.

6. Xie C, Mao X, Huang J, Ding Y, Wu J, Dong S, Kong L, Gao G, Li C-Y, Wei L: **KOBAS 2.0: a web server for annotation and identification of enriched pathways and diseases**. *Nucleic acids research* 2011, **39**(suppl_2):W316-W322.
